# Eficácia de um Substituto do Sal na Incidência de Hipertensão: Uma Revisão Sistemática com Metanálise

**DOI:** 10.36660/abc.20250440

**Published:** 2026-04-01

**Authors:** Francinny Alves Kelly, Clara Rocha Dantas, Luis Eduardo Rodrigues Sobreira, Artur Menegaz de Almeida, Fernando Baía Bezerra, Márcio Gonçalves de Sousa, Fernanda Consolim, Antonio Gabriele Laurinavicius

**Affiliations:** 1 Cardiovascular Division Beth Israel Deaconess Medical Center Boston Massachusetts EUA Cardiovascular Division, Beth Israel Deaconess Medical Center, Boston, Massachusetts – EUA; 2 Universidad de Buenos Aires Buenos Aires Argentina Universidad de Buenos Aires, Buenos Aires – Argentina; 3 Universidade Federal do Pará Altamira PA Brasil Universidade Federal do Pará (UFPA), Altamira, PA – Brasil; 4 Universidade Federal de Mato Grosso do Sul Campo Grande MS Brasil Universidade Federal de Mato Grosso do Sul, Campo Grande, MS – Brasil; 5 Universidade Anhembi Morumbi São José dos Campos SP Brasil Universidade Anhembi Morumbi (UAM), São José dos Campos, SP – Brasil; 6 Instituto Dante Pazzanese de Cardiologia São Paulo SP Brasil Instituto Dante Pazzanese de Cardiologia, São Paulo, SP – Brasil; 7 Instituto do Coração do Hospital das Clínicas Faculdade de Medicina Universidade de São Paulo São Paulo SP Brasil Instituto do Coração do Hospital das Clínicas da Faculdade de Medicina da Universidade de São Paulo, São Paulo, SP – Brasil

**Keywords:** Doenças Cardiovasculares, Pressão Arterial, Pressão Arterial Sistólica, Hipertensão

## Abstract

**Fundamento:**

Um substituto do sal enriquecido com potássio, no qual parte do cloreto de sódio é substituída por cloreto de potássio, tem demonstrado considerável potencial como estratégia em nível populacional para reduzir a ingestão de sódio e prevenir doenças cardiovasculares. Nos últimos anos, as pesquisas têm se concentrado principalmente em indivíduos com hipertensão, demonstrando que os substitutos do sal podem influenciar a pressão arterial (PA).

**Objetivo:**

Realizar uma metanálise para quantificar a magnitude da redução da PA em pacientes com hipertensão que utilizam sal comum em comparação com aqueles que utilizam um substituto do sal.

**Métodos:**

PubMed, Scopus e Web of Science foram pesquisados em busca de ensaios clínicos randomizados (ECRs) que compararam sal comum com um substituto do sal. Diferenças médias (DM) com intervalos de confiança (ICs) de 95% foram calculadas utilizando um modelo de efeitos aleatórios. A heterogeneidade foi avaliada por meio da estatística I^2^. Um valor de p < 0,05 foi considerado estatisticamente significativo.

**Resultados:**

Quatro ECRs envolvendo 1.430 participantes foram incluídos, dos quais 725 (49,57%) receberam o substituto do sal. O uso do substituto do sal foi associado a uma redução significativa da PA sistólica (PAS) (DM, −5,75 mmHg; IC 95%, −6,98 a −2,39 mmHg; I^2^ = 37%; p < 0,01) e a uma redução significativa da PA diastólica (PAD) (DM, −1,62 mmHg; IC 95%, −2,34 a −0,91 mmHg; I^2^ = 0%; p < 0,001).

**Conclusão:**

Em pacientes com hipertensão, o uso de um substituto do sal está associado a uma redução significativa tanto da PAS quanto da PAD em comparação com o sal comum.

## Graphical abstract

**Figura Central:**
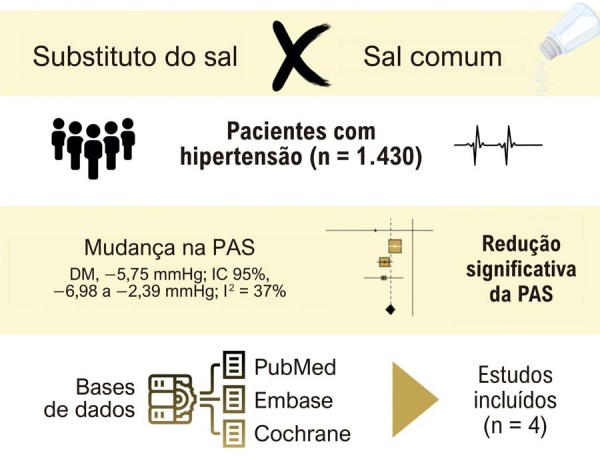
Eficácia de um Substituto do Sal na Incidência de Hipertensão: Uma Revisão Sistemática com Metanálise

DM: diferença média; PAS: pressão arterial sistólica.

## Introdução

As doenças cardiovasculares (DCV) são as condições cardíacas não transmissíveis mais comuns em todo o mundo, sendo responsáveis por aproximadamente um terço de todas as mortes globais.^1^ Nos Estados Unidos, estima-se que 62 milhões de pessoas tenham diagnóstico de DCV. Nos últimos anos, o estudo *Global Burden of Disease* (GBD) relatou que a cardiopatia hipertensiva ocupou a vigésima segunda posição entre as principais causas de morte em indivíduos com idade entre 50 e 74 anos.^2^ Em nível mundial, cerca de 62% dos casos de doença cerebrovascular e 49% dos casos de doença cardíaca isquêmica podem ser atribuídos ao aumento da pressão arterial (PA).^3^ A ingestão excessiva de sódio é um fator de risco causal para hipertensão, e a redução do sódio proveniente do sal alimentar é recomendada como tratamento de primeira linha para hipertensão.^4 - 6^ Isso levou a um debate contínuo sobre se os níveis atuais de consumo de sal são excessivamente elevados do ponto de vista da saúde e se podem desempenhar um papel importante na elevação da PA.

A Organização Mundial da Saúde propôs que uma redução de 30% na ingestão de sal pode diminuir o risco de hipertensão.^7^ Além disso, o *Centers for Disease Control and Prevention* recomenda uma ingestão diária de sódio não superior a 2.300 mg.^8^ No entanto, o consumo de sódio nos Estados Unidos permanece elevado, com média de 3.330 mg/dia. Recentemente, um número crescente de países adotou estratégias nacionais de redução do sal, incluindo a substituição do sal comum por substitutos do sal, geralmente compostos por 65% de cloreto de sódio, 25% de cloreto de potássio e 10% de sulfato de magnésio.^9 , 10^ Essa formulação constitui uma alternativa com baixo teor de sódio, comercializada para reduzir a ingestão de sal e auxiliar na prevenção e no controle da PA elevada, por meio da redução da PA sistólica (PAS) e da PA diastólica (PAD) sem alterar o sabor.^10 - 13^ Ainda assim, persiste controvérsia significativa sobre se o consumo atual de sal é excessivamente alto do ponto de vista da saúde.

Uma metanálise anterior envolvendo indivíduos normotensos indicou que a suplementação oral de potássio pode reduzir significativamente a PA, seja a PAS ou a PAD.^14^ No entanto, estudos mais recentes focados exclusivamente em pacientes hipertensos também demonstraram o efeito dos substitutos do sal na redução da PAS e da PAD. Dessa forma, esta metanálise tem como objetivo avaliar a redução da PA em pacientes com hipertensão e analisar a eficácia de um substituto do sal em comparação com o sal comum.

## Métodos

### Protocolo e registro

Esta metanálise foi conduzida de acordo com as diretrizes *Preferred Reporting Items for Systematic Reviews and Meta-Analyses* .^15^ A revisão foi registrada no *Prospective International Registry of Systematic Reviews* sob o código de registro CRD42024568555.

### Critérios de elegibilidade

Para esta revisão sistemática, estudos originais foram selecionados com base em critérios de elegibilidade previamente definidos. Apenas estudos publicados em periódicos revisados por pares que incluíram pacientes adultos (≥ 18 anos) com hipertensão confirmada foram incluídos. Os estudos elegíveis deveriam comparar o sal comum com um substituto do sal e relatar alterações na PA como desfecho primário. Apenas artigos completos publicados em inglês foram considerados.

Os critérios de exclusão foram populações sobrepostas, ausência de grupo controle ou placebo, ensaios clínicos não randomizados (ECRs), relatos de caso, revisões, artigos de opinião, relatórios técnicos, diretrizes, estudos em animais e experimentos in vitro. Apenas artigos publicados em inglês foram incluídos, sem restrições quanto ao ano de publicação.

A questão central que norteou esta revisão sistemática foi: “Em pacientes com hipertensão, como o uso de um substituto do sal impacta a PA em comparação com o sal comum?”. Essa questão foi estruturada utilizando o modelo PICOT, no qual a população (P) consistiu em pacientes com hipertensão; a exposição (E) foi definida como o uso de sal comum; o grupo de comparação (C) incluiu pacientes que utilizaram um substituto do sal; o desfecho (O) foi a mudança na PAS e na PAD; e o delineamento do estudo (T) foi limitado a ECRs.

### Estratégia de Busca

Uma busca sistemática da literatura foi realizada nas bases PubMed, Web of Science e Cochrane Central desde a criação das bases até a data da busca final. A estratégia foi elaborada para identificar estudos que avaliassem os efeitos de substitutos do sal ou sais com teor reduzido de sódio na incidência de hipertensão. A busca combinou vocabulário controlado ( *Medical Subject Headings* [MeSH]) e termos livres relacionados a substitutos do sal (por exemplo, “salt substitutes”, “low-sodium salt”, “reduced sodium salt”, “potassium-enriched salt”, “dietary sodium”) e hipertensão (por exemplo, “hypertension”, “high blood pressure”, “incidence”, “new-onset hypertension”, “development of hypertension”), utilizando operadores booleanos (OR, AND), conforme segue:

(“Salt Substitutes”[MeSH] OR “Dietary Sodium”[MeSH] OR “Potassium, Dietary”[MeSH] OR salt substitute OR low sodium salt OR reduced sodium salt OR potassium-enriched salt)* AND (“Hypertension”[MeSH] OR hypertension OR high blood pressure) AND (“Incidence”[MeSH] OR incidence OR new-onset hypertension OR development of hypertension).

Estratégias de busca equivalentes foram adaptadas para Web of Science e Cochrane Central, utilizando a sintaxe e o vocabulário controlado específicos de cada base. Resumos, textos completos e anais de congressos científicos foram avaliados. As listas de referências dos estudos incluídos e de revisões sistemáticas relevantes foram revisadas manualmente para identificar estudos adicionais elegíveis.

A triagem inicial foi baseada em títulos e resumos, sem restrições quanto à data de publicação. Todos os registros recuperados foram importados para a plataforma Rayyan^®^ para facilitar o processo de seleção.^16^ Nessa etapa, estudos não relacionados à pergunta de pesquisa foram excluídos e duplicatas foram removidas. Três autores (C.R.D, L.E.R.S e F.B.B) realizaram a triagem de forma independente, e divergências foram resolvidas por meio de discussão com um terceiro autor (F.A.K). Alertas automáticos também foram configurados nas bases de dados para notificar a equipe sobre novos estudos publicados durante o período de análise.

### Extração de dados

Para sintetizar os principais achados, três autores revisores (F.A.K, L.E.R.S e F.B.B) extraíram de forma independente os dados dos quatro artigos incluídos. As informações coletadas incluíram sobrenome do primeiro autor, ano de publicação, delineamento do estudo, período de seguimento, características dos pacientes (idade, sexo e comorbidades), métodos de avaliação e conclusões quanto às mudanças na PAS e na PAD. Divergências foram resolvidas por consenso, e um terceiro autor revisor (C.R.D) tomou a decisão final quando necessário.

### Desfechos e Definições

O desfecho primário de interesse foi a mudança na PAS e na PAD.

### Avaliação do Risco de Viés

A ferramenta *Revised Cochrane Risk-of-Bias Tool for Randomized Trials* ^17^ foi utilizada para avaliar a qualidade metodológica dos ECRs incluídos. Dois autores (C.R.D e L.E.R.S) avaliaram todos os estudos de forma independente, e divergências foram resolvidas por consenso. Cada ensaio foi classificado como tendo risco de viés alto, baixo ou incerto em cinco domínios: processo de randomização, desvios das intervenções pretendidas, dados de desfecho ausentes, mensuração dos desfechos e seleção dos resultados relatados.

O potencial viés de publicação e efeitos de estudos pequenos foram avaliados por meio da análise de gráfico de funil, a fim de determinar se a distribuição dos ensaios era simétrica.

### Análise Estatística

Os efeitos do tratamento para desfechos contínuos foram agrupados e expressos como diferenças médias (DM) com intervalos de confiança (ICs) de 95%. A heterogeneidade foi avaliada pela amplitude dos tamanhos de efeito e pelas estatísticas I^2^ e τ^2^. Um modelo de efeito fixo foi utilizado para desfechos com I^2^ < 25%, enquanto o modelo de efeitos aleatórios de DerSimonian e Laird foi aplicado aos desfechos combinados com maior heterogeneidade, considerando tanto o I^2^ quanto o τ^2^ (variância entre estudos). Um valor de p < 0,05 foi considerado estatisticamente significativo.

As análises estatísticas foram realizadas utilizando o RStudio versão 4.2.3, com o pacote “metafor” (R Foundation for Statistical Computing). Uma análise de sensibilidade foi conduzida para avaliar o impacto de estudos individuais, removendo sequencialmente cada ECR e reanalisando os dados restantes (análise *leave-one-out* ). A dominância do estudo foi definida quando a remoção de um estudo alterava a significância estatística dos valores de p do tamanho de efeito combinado, seja de significativo para não significativo ou vice-versa.

## Resultados

### Seleção de estudos e características basais

**FIgura 1:**
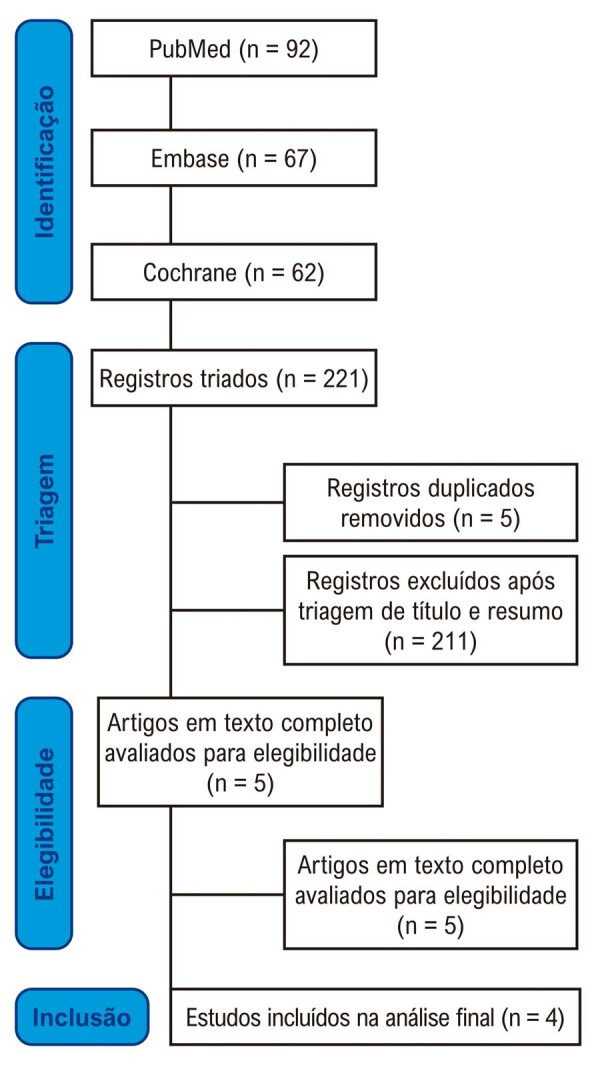
– Diagrama de fluxo do Preferred Reporting Items for Systematic Reviews and Meta-Analyses resumindo o processo de identificação, triagem, elegibilidade e inclusão dos estudos.

Conforme detalhado na Figura 1 , a busca inicial identificou 221 registros. Após a remoção de duplicatas e a triagem dos estudos com base em título e resumo, cinco estudos permaneceram para revisão do texto completo, de acordo com os critérios pré-estabelecidos. Destes, quatro estudos foram incluídos, totalizando 1.430 pacientes, dos quais 725 (50,6%) receberam o substituto do sal. A estratégia de busca está resumida na Figura 1 . A Figura Central apresenta os principais achados deste estudo.

Entre os quatro estudos incluídos, foram analisados 1.430 pacientes, com 725 (50,6%) recebendo o substituto do sal e 705 (49,3%) recebendo sal comum. O tempo de seguimento variou de 2 semanas a 2 anos. A população do estudo incluiu 777 (54,3%) homens e 653 (45,6%) mulheres; 292 (20,4%) tinham histórico de tabagismo e 303 (21,1%) relataram consumo de álcool. A idade média variou de 61,5 a 71,8 anos, o índice de massa corporal (IMC) variou de 23,1 a 31 kg/m^2^, a pressão arterial sistólica (PAS) variou de 121,9 a 177,6 mmHg e a pressão arterial diastólica (PAD) variou de 74,4 a 105,8 mmHg. As características basais de cada estudo incluído são apresentadas na Tabela 1 .

**Tabela 1:** – Características demográficas, clínicas e de tratamento basais dos estudos incluídos, apresentadas por estudo

Característica	Barros et al.^**18**^	Zhao et al.^**19**^	Yu et al.^**20**^	Zhang et al.^**21**^
**Número de pacientes**	GI: 19; CG: 16	GI: 141; CG: 141	GI: 252; CG: 250	GI: 313; GC: 298
**Idade, anos (DP)**	—	GI: 62,8 (11,1); CG: 63,5 (11,3)	GI: 61,5 (11,1); CG: 61,7 (12,9)	GI: 71 (9,7); GC: 71,8 (10,3)
**Seguimento**	4 semanas	3 meses	3 meses	2 anos
**Sexo masculino, n (%)**	—	GI: 56 (39,7%); CG: 60 (42,6%)	GI: 105 (41,7%); CG: 102 (40,8%)	GI: 231 (73,8%); GC: 223 (74,8%)
**IMC, kg/m^**2**^ (DP)**	GI: 29,38 (5,55); CG: 31 (5,97)	GI: 23,7 (3,1); CG: 23,6 (3,4)	GI: 23,1 (4,7); CG: 23,6 (4,2)	—
**Histórico de tabagismo, n (%)**	—	GI: 6 (8,2%); CG: 7 (9,7%)	GI: 37 (14%); CG: 34 (13,6%)	GI: 110 (35,1%); GC: 98 (32,9%)
**Histórico de etilismo, n (%)**	—	GI: 8 (11%); CG: 9 (12,5%)	GI: 115 (45,6%); CG: 110 (44%)	GI: 33 (10,5%); GC: 28 (9,4%)
**PAS, mmHg (DP)**	GI: 142,95 (14,86); CG: 143,44 (13,99)	GI: 176,1 (22,4); CG: 177,6 (23,3)	GI: 132,8 (20,3); CG: 132,1 (22,5)	GI: 122 (12); GC: 121,9 (11,1)
**PAS, mmHg (DP)**	GI: 89,79 (9,10); CG: 91,19 (9,10)	GI: 103,2 (12); CG: 105,8 (13,1)	GI: 83,7 (12); CG: 82,9 (13,1)	GI: 74,4 (8,5); GC: 74,5 (7,7)
**Uso de anti-hipertensivos, n (%)**	—	GI: 61 (47%); CG: 71 (50,7%)	Diuretic = GI: 0; CG: 1 (0,4%) CCB = GI: 6 (2,4%); CG: 9 (3,6%) ACEi or ARB = GI: 70 (27,8%); CG: 80 (32%) β-blocker = GI: 63 (25%); CG: 50 (20%) α-blocker = GI: 107 (42,5%); CG: 97 (39%)	—
**Composição do substituto do sal**	GI: NaCl 27,3% + KCl 72,6%; CG: NaCl 100%	GI: NaCl 70% + KCl 30% + MgSO_4_ 10%; CG: NaCl 100%	GI: NaCl 70% + KCl 30%; CG: NaCl 100%	GI: NaCl 62,5% + KCl 25% + 12,5% de aromatizantes; CG: NaCl 100%
**Histórico de DCV, n (%)**	—	—	GI: 3 (1,2%); CG: 4 (1,6%)	GI: 56 (17,9%); CG: 67 (22,5%)
**Histórico de diabetes, n (%)**	—	—	GI: 59 (23,4%); CG: 51 (20,5%)	—

Todos os estudos incluídos adotaram nível de significância estatística de 5% (α = 0,05). BRA: bloqueador do receptor de angiotensina II; DCV: doença cardiovascular; DP: desvio padrão; GC: grupo controle; GI: grupo intervenção; iECA: inibidor da enzima conversora de angiotensina; PA: pressão arterial.

### Mudanças na pressão arterial sistólica

**Figura 2:**
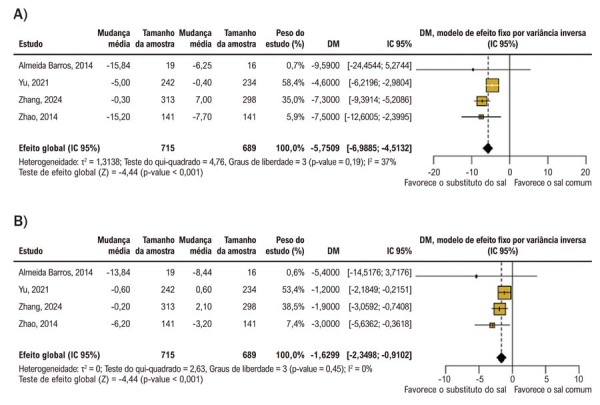
– Forest plots da DM nas mudanças da PA comparando substituto do sal versus sal comum. A) Mudança na PAS; B) Mudança na PAD. DM: diferença média; IC: intervalo de confiança; PA: pressão arterial; PAD: PA diastólica; PAS: PA sistólica.

A PAS foi significativamente reduzida no grupo que utilizou o substituto do sal em comparação com o grupo que utilizou sal comum (DM, −5,75 mmHg; IC 95%, −6,98 a −2,39 mmHg; I^2^ = 37%; τ^2^ = 1,31; p < 0,01) ( Figura 2A ).

A substituição do sal resultou em uma redução aproximada de 5 mmHg na PAS entre os estudos incluídos. Reduções dessa magnitude têm sido associadas a uma diminuição de 10%-15% no risco de eventos cardiovasculares maiores, incluindo acidente vascular cerebral e infarto do miocárdio, sugerindo um potencial impacto populacional sobre o risco cardiovascular.

### Mudanças na pressão arterial diastólica

A PAD foi significativamente reduzida no grupo que utilizou o substituto do sal em comparação com o grupo que utilizou sal comum (DM, −1,62 mmHg; IC 95%, −2,34 a −0,91 mmHg; I^2^ = 0%; τ^2^ = 0; p < 0,001) ( Figura 2B ).

Embora a redução na PAD tenha sido menor, mesmo diminuições modestas na PAD têm sido associadas a menor risco de eventos cardiovasculares, especialmente em adultos mais velhos. Isso sugere que os substitutos do sal podem contribuir para a redução global do risco cardiovascular, complementando os benefícios observados para a PAS.

Os efeitos específicos por subgrupos não puderam ser avaliados, pois nenhum dos estudos incluídos relatou os desfechos separadamente para populações de alto risco, como indivíduos com doença renal crônica ou DCV estabelecida. Embora as reduções observadas na PAS (−5,75 mmHg) e na PAD (−1,62 mmHg) sejam clinicamente relevantes em nível individual, as implicações subsequentes para a prevenção cardiovascular em larga escala permanecem incertas. Estudos de modelagem em grande escala demonstraram que até mesmo pequenas reduções médias na PA podem levar a reduções significativas na morbidade e mortalidade cardiovascular; no entanto, esta metanálise não dispôs de dados suficientes para estimar diretamente esses benefícios. São necessários ECRs adicionais, com análises estratificadas e seguimento mais prolongado, para esclarecer o real impacto em saúde pública das intervenções com substitutos do sal.

### Avaliação do risco de viés

A Figura Suplementar Online 1 e a Figura Suplementar Online 2 resumem o risco de viés de cada estudo incluído. Todos os quatro estudos foram classificados como apresentando baixo risco de viés, resultando em um risco global de viés considerado baixo para a análise.

**Figura 5:**
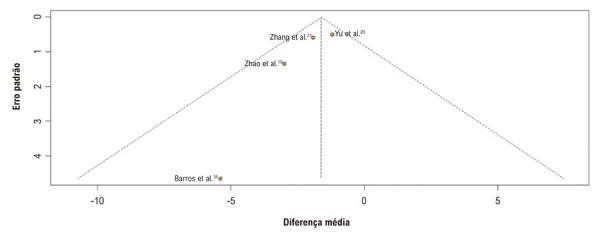
– Funnel plot para avaliação de viés de publicação na análise.

Os gráficos de funil para os desfechos mostraram uma distribuição assimétrica dos estudos incluídos ( Figura 5 ; Figura Suplementar Online 3 ).

### Análise de Sensibilidade

Nenhum estudo foi excluído devido à heterogeneidade metodológica; análises de sensibilidade do tipo *leave-one-out* e gráficos de Baujat foram realizadas para todos os desfechos contínuos.

**Figura 3:**
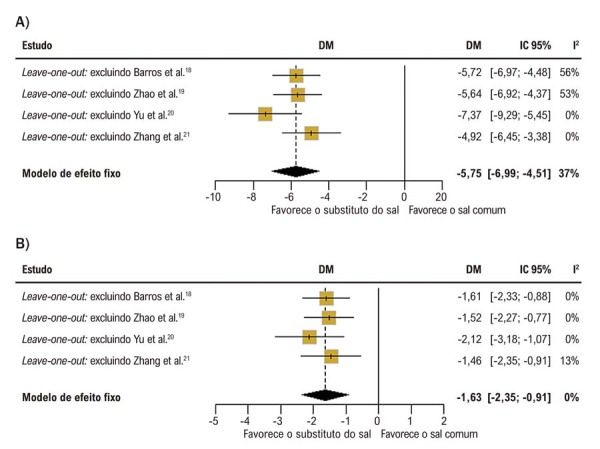
– Forest plots da análise de sensibilidade leave-one-out para a DM da PA comparando substituto do sal versus sal comum. A) Mudança na PAS; B) Mudança na PAD. DM: diferença média; IC: intervalo de confiança; PA: pressão arterial; PAD: PA diastólica; PAS: PA sistólica.

Na análise *leave-one-out* para PAS, a exclusão de Barros et al.^18^ e Zhao et al.^19^ aumentou a heterogeneidade para 56% e 53%, respectivamente. Em contraste, a exclusão de Yu et al.^20^ e Zhang et al.^21^ reduziu a heterogeneidade para 0% em ambas as análises ( Figura 3A ). Para PAD, não houve mudança significativa na heterogeneidade com a remoção individual dos estudos; no entanto, quando Zhang et al.^21^ foi excluído, a heterogeneidade aumentou para 13% ( Figura 3B ).

**Figura 4:**
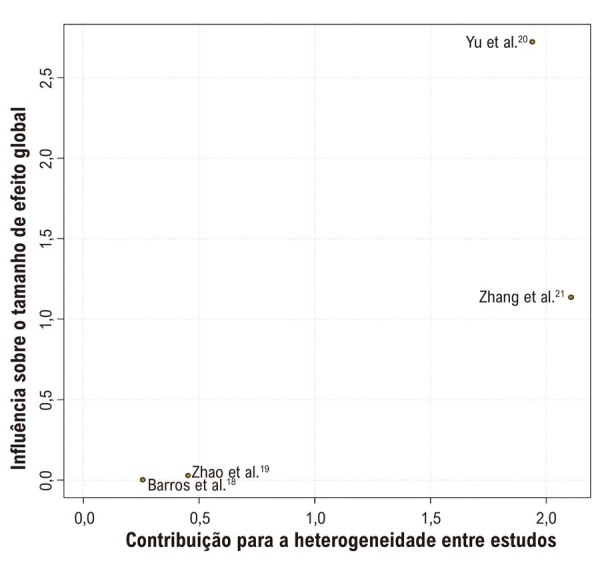
– Gráfico de Baujat para pressão arterial sistólica.

A análise por gráfico de Baujat indicou que Yu et al.^20^ exerceu a maior influência sobre os resultados globais tanto para PAS quanto para PAD. Além disso, Zhang et al.^21^ foi o que mais contribuiu para a heterogeneidade geral em ambas as análises ( Figura 4 ; Figura Suplementar 4 ).

### Análise de metarregressão

Foi realizada uma metarregressão para investigar se a idade média e o IMC médio poderiam explicar a heterogeneidade entre os estudos nos desfechos relacionados à PA. Para a PAS, a idade apresentou associação limítrofe com o efeito do tratamento, sugerindo uma tendência a maior redução da PA em estudos que incluíram participantes mais idosos (coeficiente: −0,26; p = 0,0557; I^2^ = 0%). Em contraste, o IMC não se associou significativamente à resposta sistólica à intervenção (coeficiente: −0,84; p = 0,4535; I^2^ = 0%).

Para a PAD, nem a idade (coeficiente: −0,04; p = 0,7387; I^2^ = 29,9%) nem o IMC (coeficiente: −0,67; p = 0,3388; I^2^ = 19,3%) foram moderadores significativos do efeito do tratamento. Esses achados sugerem que os efeitos redutores da PA dos substitutos do sal parecem consistentes independentemente da idade ou do IMC dos participantes dentro das faixas relatadas nos ensaios incluídos ( Figura Suplementar Online 5 ; Figura Suplementar Online 6 ; Figura Suplementar Online 7 ; Figura Suplementar Online 8 ).

## Discussão

Nesta revisão sistemática com metanálise de quatro ECRs incluindo 1.430 participantes, os substitutos do sal, geralmente formulados por meio da substituição parcial do cloreto de sódio por cloreto de potássio e/ou cálcio, foram associados a reduções significativas da PA em comparação com o sal comum. A hipertensão é o principal fator de risco global e contribui para a perda de 174 milhões de anos de vida ajustados por incapacidade anualmente,^22^ e seu manejo permanece subótimo em países de baixa e média renda. Como o sal adicionado durante o preparo dos alimentos responde pela maior parte da ingestão de sódio nesses contextos, os substitutos do sal podem representar uma intervenção viável e de grande impacto.^23 , 24^

A redução combinada na PAS foi de −5,8 mmHg (IC 95%, −7 a −4,6 mmHg), enquanto a PAD diminuiu −1,6 mmHg (IC 95%, −2,3 a −0,9 mmHg). Esses achados demonstram um efeito consistente de redução da PA, comparável em magnitude ao de intervenções anti-hipertensivas estabelecidas, e sustentam a substituição do sal como uma estratégia promissora para o controle populacional da PA.

Nossos achados integram e ampliam pesquisas anteriores que avaliaram os efeitos dos substitutos do sal sobre a PA. Investigações iniciais conduzidas antes de 2010 forneceram evidências limitadas de reduções clinicamente relevantes da PA.^21 , 25 - 29^ Uma exceção importante foi o ECR por conglomerados realizado na China por Mu et al.,^24^ que demonstrou uma redução de 4,2/1,9 mmHg na PAS e na PAD ao longo de 12 semanas. A redução da PAS observada em nossa análise combinada (−5,8 mmHg) é amplamente consistente com esse sinal inicial e reforça que o efeito hipotensor dos substitutos do sal persiste entre diferentes faixas etárias e contextos culturais.

Ensaios clínicos mais recentes em adultos relataram reduções na PAS de aproximadamente 3-6 mmHg, valores muito próximos da magnitude estimada neste estudo. Barros et al.^18^ observaram uma redução adicional de 5,6 mmHg na PAS com uma preparação contendo 50% de NaCl/50% de KCl ao longo de 3 meses, enquanto Zhao et al.^19^ relataram uma diminuição de 4,8/1,7 mmHg após 6 meses. De forma semelhante, Yu et al.^20^ e Zhang et al.^21^ documentaram reduções da PAS de 3-5 mmHg. A consistência entre nossos achados e esses ensaios reforça a robustez do tamanho de efeito e destaca sua reprodutibilidade em diferentes regiões geográficas, perfis de risco basal e formulações de sal enriquecido com potássio.

Além disso, a magnitude da redução da PAS observada nesta metanálise está alinhada com modelos epidemiológicos em grande escala derivados do estudo *INTERnational study of SALT and blood pressure* e de estudos do GBD,^30 - 33^ que associam reduções populacionais de aproximadamente 5 mmHg na PAS a diminuições significativas do risco cardiovascular. Embora essas estimativas forneçam contexto para possíveis benefícios em saúde pública, elas permanecem extrapolações teóricas, e não desfechos diretamente mensurados nos ensaios incluídos.

Nossos achados reforçam o potencial dos substitutos do sal como uma estratégia eficaz, baseada no ambiente familiar, para a prevenção primária da hipertensão.^34 , 35^ A substituição do cloreto de sódio por cloreto de potássio reduz o volume extracelular ao mesmo tempo em que aumenta a natriurese e a vasodilatação.^36 - 39^ Do ponto de vista da saúde pública, os substitutos do sal são particularmente adequados para países de baixa e média renda, onde o consumo de sal adicionado durante o preparo dos alimentos representa mais de 70% da ingestão total e a reformulação industrial permanece limitada.^39 - 43^ A combinação da substituição do sal com estratégias educativas voltadas à redução do consumo total para ≤ 3 g/dia pode ampliar ainda mais o benefício.^43 - 45^ Nesta metanálise, os substitutos do sal enriquecidos com potássio reduziram a PAS com uma DM combinada de −5,8 mmHg (IC 95%, −7 a −4,6 mmHg) e a PAD em −1,6 mmHg (IC 95%, −2,3 a −0,9 mmHg) em comparação com o sal comum. Essas reduções são comparáveis às alcanadas com medicamentos anti-hipertensivos de primeira linha.

Apesar desses achados encorajadores, diversas limitações metodológicas dos estudos incluídos devem ser reconhecidas. Primeiro, a heterogeneidade entre os estudos, particularmente para a PAS, foi moderada, refletindo diferenças nas populações de pacientes, na duração do seguimento e nas composições das intervenções. Segundo, alguns ensaios apresentaram períodos de intervenção curtos, limitando a avaliação da manutenção da redução da PA e da segurança em longo prazo. Terceiro, o viés de publicação não pode ser completamente excluído devido ao pequeno número de ECRs e aos critérios de inclusão relativamente restritos. Quarto, embora todos os estudos incluídos tenham sido randomizados, os procedimentos de cegamento e o relato de adesão variaram, podendo introduzir viés de desempenho ou de detecção. Por fim, o monitoramento do potássio sérico foi relatado de forma inconsistente, o que gera incerteza quanto à segurança em longo prazo dos sais enriquecidos com potássio, especialmente em relação ao risco de hipercalemia em pacientes com função renal comprometida ou em uso de fármacos bloqueadores do sistema renina-angiotensina-aldosterona.

## Conclusão

Esta metanálise demonstra que o uso de substitutos do sal enriquecidos com potássio é significativamente mais eficaz do que o sal comum na redução tanto da PAS quanto da PAD em pacientes com hipertensão. Esses achados reforçam o papel dos substitutos do sal como uma estratégia prática e de baixo custo para a prevenção e o manejo da hipertensão, com potencial para reduzir substancialmente eventos cardiovasculares em nível populacional.

Entretanto, questões importantes permanecem em aberto, particularmente em relação à segurança em longo prazo dessas intervenções e aos seus efeitos hipotensores em indivíduos normotensos, que frequentemente foram excluídos dos ECRs. Estudos clínicos futuros, em larga escala, devem incluir essas populações, incorporar monitoramento sistemático do potássio e explorar os mecanismos subjacentes aos efeitos redutores da PA promovidos pelos substitutos do sal. Tais investigações serão essenciais para caracterizar plenamente seu potencial preventivo e para orientar estratégias de precisão no cuidado cardiovascular.

De modo geral, esta metanálise sustenta o papel dos substitutos do sal como uma ferramenta acessível e custo-efetiva para reduzir a PA e potencialmente prevenir eventos cardiovasculares. Ainda assim, para garantir uma implementação segura, são necessários ECRs futuros com seguimento mais prolongado e monitoramento padronizado do potássio, a fim de confirmar a durabilidade dos efeitos, otimizar as proporções de composição e avaliar desfechos em longo prazo, incluindo hipercalemia e mortalidade cardiovascular.

## Material suplementar

Supplementary Material
