# Supplementary material for: Eficácia de um Substituto do Sal na Incidência de Hipertensão: Uma Revisão Sistemática com Metanálise
Source: Arq Bras Cardiol. 2026 Apr 1;123(3):e20250440. [Article in Portuguese] doi: 10.36660/abc.20250440 (PMC13128219; doi:10.36660/abc.20250440)
Supplement: Supplementary Material [file 0066-782x-abc-123-3-e20250440-suppl01.pdf]

Supplementary Material

Online Resource

Efficacy of a Salt Substitute on Incidence of Hypertension: a Systematic Review and Meta-Analysis

Online Resource Figure 1 and Figure 2. Risk of bias summary for randomized studies (RoB 2): Critical appraisal of individual studies according to the Cochrane Collaboration's tool for assessing risk of bias in randomized trials.

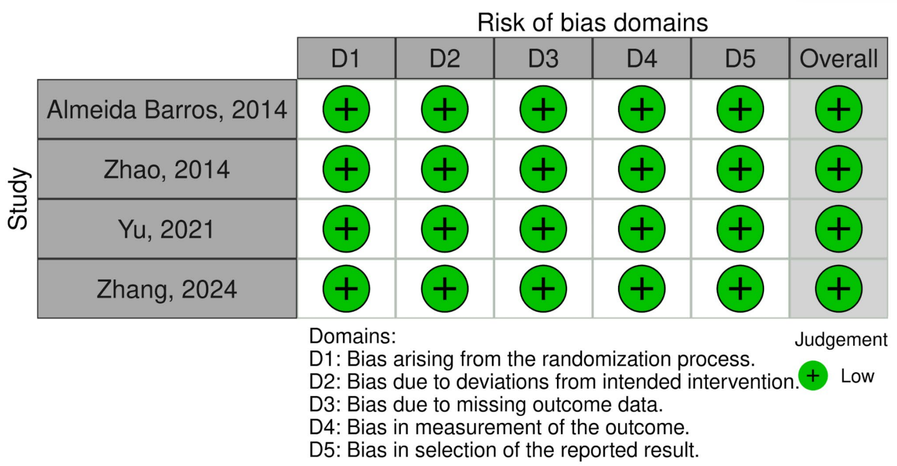

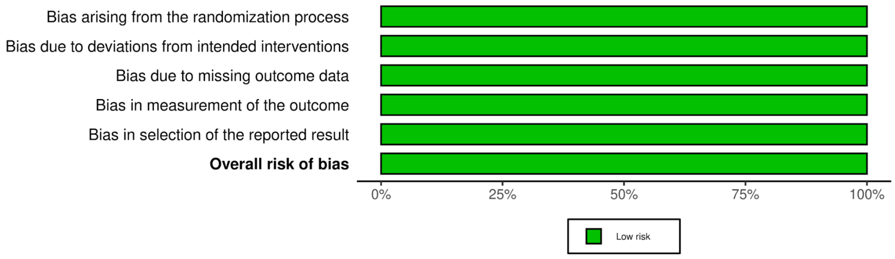

Sterne JAC, Savović J, Page MJ, Elbers RG, Blencowe NS, Boutron I, Cates CJ, Cheng H, Corbett MS, Eldridge SM, Hernán MA, Hopewell S, Hróbjartsson A, Junqueira DR, Jüni P, Kirkham JJ, Lasserson T, Li T, McAleenan A, Reeves BC, Shepperd S, Shrier I, Stewart LA, Tilling K, White IR, Whiting PF, Higgins JPT. RoB 2: a revised tool for assessing risk of bias in randomised trials. *BMJ* 2019; **366**: l4898.

Online Resource Figure 3. Funnel plot of analysis of Diastolic Blood Pressure.

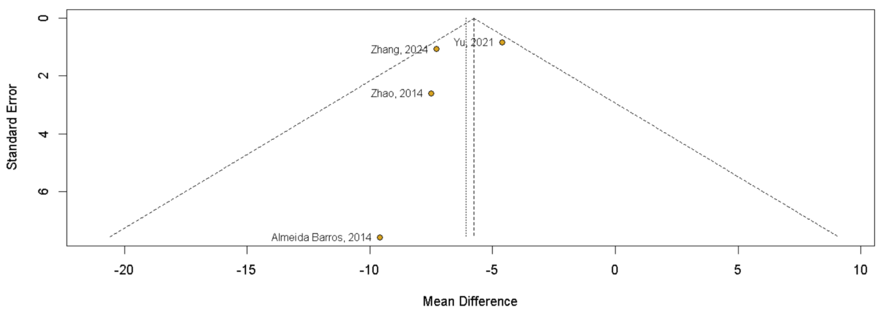

Fig. 3 Funnel Plot of Analysis of Diastolic Blood Pressure

Online Resource Figure 4. Baujat plot of analysis of Diastolic Blood Pressure.

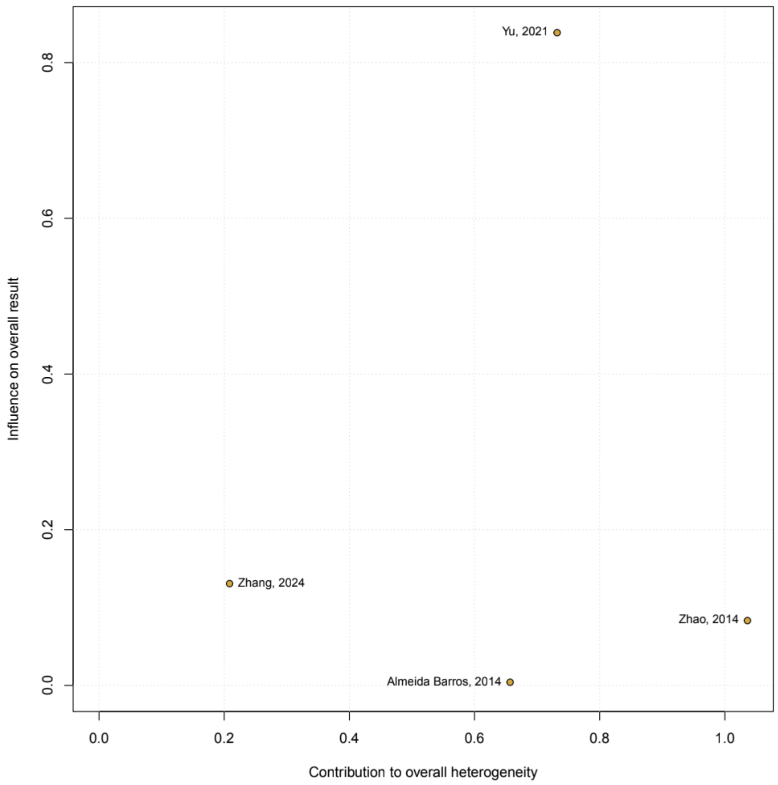

Fig. 4 Baujat Plot of Analysis of Diastolic Blood Pressure

Online Resource Figure 5, 6, 7 and 8 Meta-regression of the mean difference in systolic blood pressure and diastolic blood pressure according mean age and BMI of participants

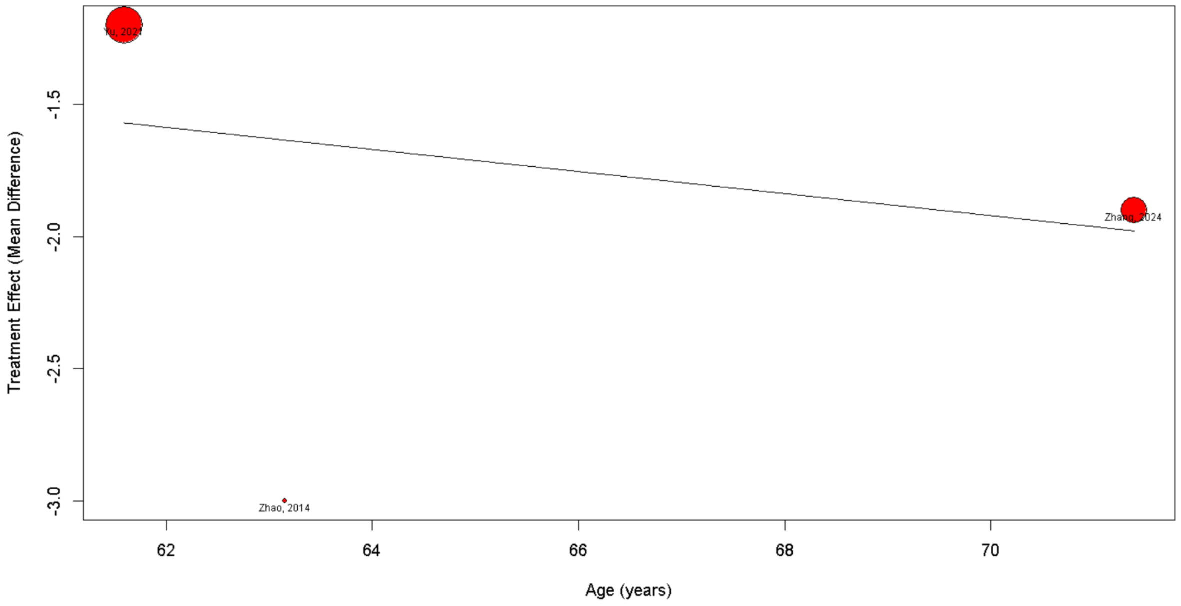

Fig 5. Meta-regression of the mean difference in diastolic blood pressure according to mean age of participants. The size of each circle reflects the study's relative weight in the analysis. The fitted line represents the estimated linear relationship between age and treatment effect.

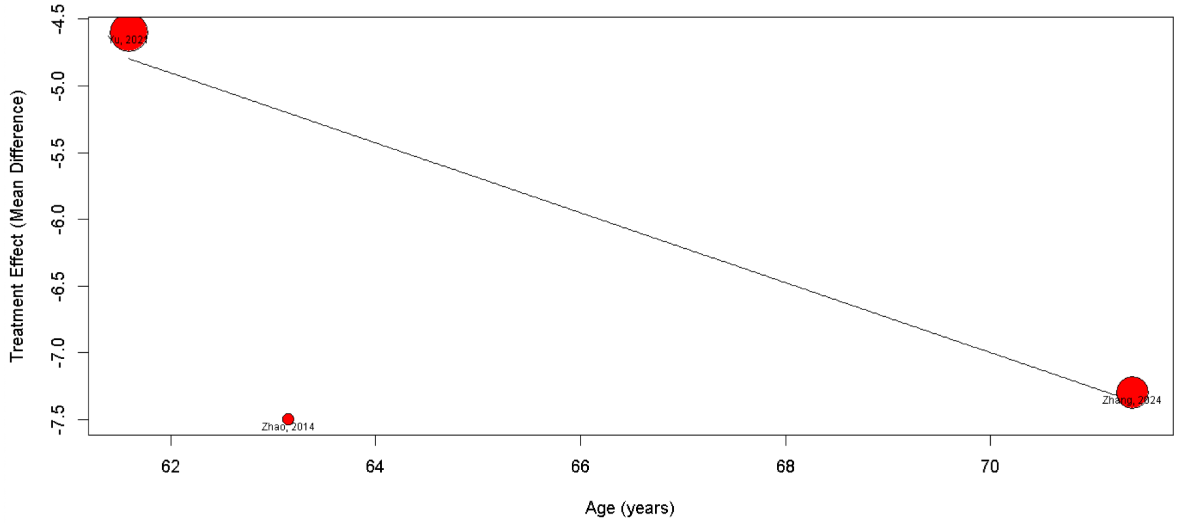

Fig 6. Meta-regression of the mean difference in systolic blood pressure according to mean age of participants. The size of each circle reflects the study's relative weight in the analysis. The fitted line represents the estimated linear relationship between age and treatment effect.

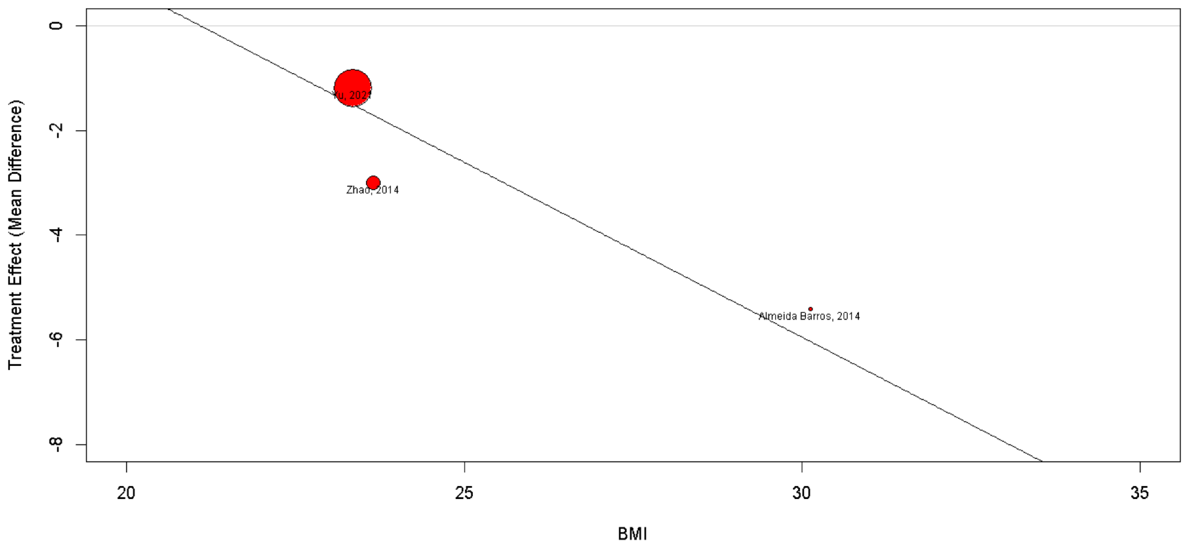

Fig 7. Meta-regression of the mean difference in diastolic blood pressure according to mean BMI of participants. The size of each circle reflects the study's relative weight in the analysis. The fitted line represents the estimated linear relationship between age and treatment effect.

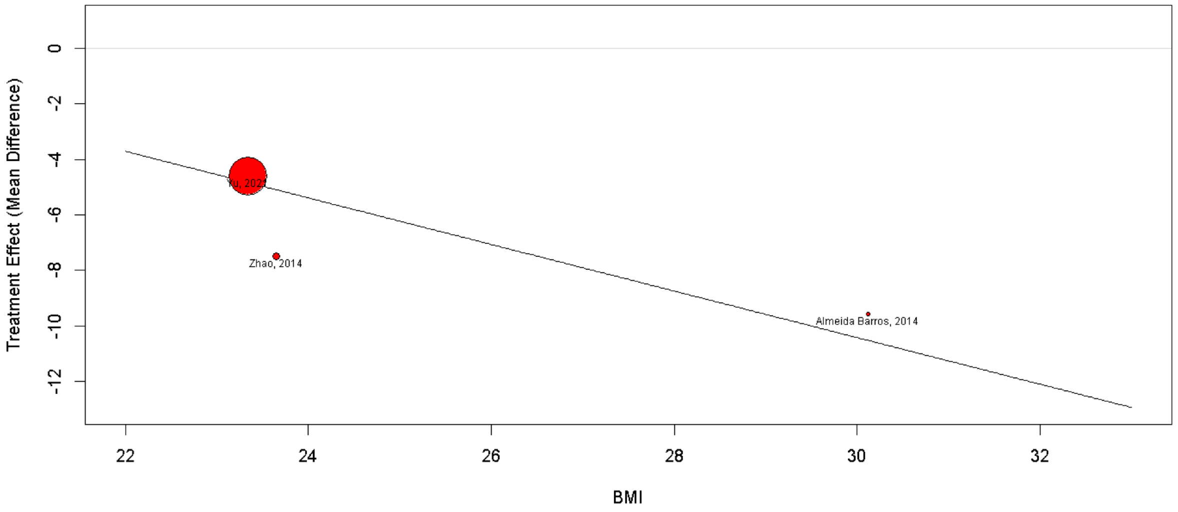

Fig 8. Meta-regression of the mean difference in systolic blood pressure according to mean BMI of participants. The size of each circle reflects the study's relative weight in the analysis. The fitted line represents the estimated linear relationship between age and treatment effect.
