# Supplementary material for: Eficácia de um Substituto do Sal na Incidência de Hipertensão: Uma Revisão Sistemática com Metanálise
Source: Arq Bras Cardiol. 2026 Apr 1;123(3):e20250440. [Article in Portuguese] doi: 10.36660/abc.20250440 (PMC13128219; doi:10.36660/abc.20250440)
Supplement: Translation

# Translation: Efficacy of a Salt Substitute on the Incidence of Hypertension: A Systematic Review with Meta-Analysis

**DOI:** 10.36660/abc.20250440i

**Authors:** Francinny Alves Kelly, Clara Rocha Dantas, Luis Eduardo Rodrigues Sobreira, Artur Menegaz de Almeida, Fernando Baía Bezerra, Márcio Gonçalves de Sousa, Fernanda Consolim, Antonio Gabriele Laurinavicius

**Affiliations:** 1 USA Cardiovascular Division, Beth Israel Deaconess Medical Center, Boston, Massachusetts – USA; 2 Argentina Universidad de Buenos Aires, Buenos Aires – Argentina; 3 Brazil Universidade Federal do Pará (UFPA), Altamira, PA – Brazil; 4 Brazil Universidade Federal de Mato Grosso do Sul, Campo Grande, MS – Brazil; 5 Brazil Universidade Anhembi Morumbi (UAM), São José dos Campos, SP – Brazil; 6 Brazil Instituto Dante Pazzanese de Cardiologia, São Paulo, SP – Brazil; 7 Brazil Instituto do Coração do Hospital das Clínicas da Faculdade de Medicina da Universidade de São Paulo, São Paulo, SP – Brazil

**Keywords:** Cardiovascular Diseases, Arterial Pressure, Hypertension

## Abstract

**Background:**

A potassium-enriched salt substitute, in which part of sodium chloride is replaced with potassium chloride, has shown considerable potential as a population-level strategy to reduce sodium intake and prevent cardiovascular disease. In recent years, research has focused primarily on individuals with hypertension, demonstrating that salt substitutes can influence blood pressure (BP).

**Objective:**

To perform a meta-analysis quantifying the magnitude of BP reduction in patients with hypertension using regular salt compared with those using a salt substitute.

**Methods:**

PubMed, Scopus, and Web of Science were searched for randomized controlled trials (RCTs) comparing regular salt with a salt substitute. Mean differences (MD) with 95% CIs were calculated using a random-effects model. Heterogeneity was assessed using the I^2^statistic. A p-value < 0.05 was considered statistically significant.

**Results:**

Four RCTs involving 1,430 participants were included, of whom 725 (49.57%) received the salt substitute. The use of a salt substitute was associated with a significant reduction in systolic BP (SBP) (MD, −5.75 mmHg; 95% CI, −6.98 to −2.39 mmHg; I^2^= 37%; p < 0.01) and a significant reduction in diastolic BP (DBP) (MD, −1.62 mmHg; 95% CI, −2.34 to −0.91 mmHg; I^2^= 0%; p < 0.001).

**Conclusion:**

In patients with hypertension, the use of a salt substitute is associated with a significant reduction in both SBP and DBP compared with regular salt.

## Introduction

Cardiovascular diseases (CVD) are the most common noncommunicable cardiac conditions worldwide, accounting for approximately one-third of all deaths globally.^1^In the United States, an estimated 62 million people are diagnosed with CVD. In recent years, the Global Burden of Disease (GBD) study reported that hypertensive heart disease ranked as the twenty-second leading cause of death among individuals aged 50-74 years.^2^Worldwide, around 62% of cerebrovascular disease and 49% of ischemic heart disease cases can be attributed to increased blood pressure (BP).^3^Excess sodium intake is a causal risk factor for hypertension, and reducing sodium from dietary salt is recommended as a first-line treatment for hypertension.^4 - 6^This has led to ongoing debate about whether current levels of salt intake are excessively high from a health perspective and may play a major role in the high BP burden.

The World Health Organization has proposed that a 30% reduction in salt intake may reduce the risk of hypertension.^7^Furthermore, the Centers for Disease Control and Prevention recommends a daily sodium intake of no more than 2,300 mg.^8^However, sodium consumption in the United States remains high, averaging 3,330 mg/day. Recently, an increasing number of countries have adopted national salt-reduction strategies, including replacing common salt with salt substitutes, typically composed of 65% sodium chloride, 25% potassium chloride, and 10% magnesium sulfate.^9 , 10^This formulation is a low-sodium salt alternative marketed to reduce salt intake and help prevent and manage high BP by lowering systolic BP (SBP) and diastolic BP (DBP) without affecting taste.^10 - 13^Nevertheless, significant controversy persists regarding whether current salt consumption is excessively high from a health perspective.

A previous meta-analysis involving normotensive subjects indicated that oral potassium supplementation can significantly reduce BP, including both SBP and DBP.^14^However, more recent studies focusing exclusively on hypertensive patients have also demonstrated the effect of salt substitutes in lowering SBP and DBP. Therefore, this meta-analysis aims to evaluate BP decrease in patients with hypertension and to assess the efficacy of a salt substitute compared with regular salt.

## Methods

### Protocol and Registration

This meta-analysis was conducted in accordance with the Preferred Reporting Items for Systematic Reviews and Meta-Analyses guidelines.^15^The review was registered in the Prospective International Registry of Systematic Reviews under the registry code CRD42024568555.

### Eligibility criteria

For this systematic review, original studies were selected based on predefined eligibility criteria. Only studies published in peer-reviewed journals that enrolled adult patients (≥ 18 years) with confirmed hypertension were included. Eligible studies were required to compare regular salt with a salt substitute and to report changes in BP as a primary endpoint. Only full-text articles published in English were considered.

Exclusion criteria were overlapping populations, absence of a control or placebo group, non-randomized clinical trials (RCTs), case reports, reviews, opinion articles, technical reports, guidelines, animal studies, and in vitro experiments. Only articles published in English were included, with no restrictions on year of publication.

The central question guiding this systematic review was: “In patients with hypertension, how does the use of a salt substitute impact BP compared with regular salt?” This question was structured using the PICOT framework, in which the population (P) consisted of patients with hypertension; the exposure (E) was defined as the use of regular salt; the comparison (C) group included patients using a salt substitute; the outcome (O) was the change in SBP and DBP; and the study design (T) was limited to RCTs.

### Search Strategy

A systematic literature search was conducted in PubMed, Web of Science, and Cochrane Central from database inception to the date of the final search. The strategy was designed to identify studies evaluating the effects of salt substitutes or reduced-sodium salts on hypertension incidence. The search combined controlled vocabulary (Medical Subject Headings [MeSH]) and free-text terms related to salt substitutes (e.g., “salt substitutes,” “low-sodium salt,” “reduced sodium salt,” “potassium-enriched salt,” “dietary sodium”) and hypertension (e.g., “hypertension,” “high blood pressure,” “incidence,” “new-onset hypertension,” “development of hypertension”) using Boolean operators (OR, AND), as follows:

(“Salt Substitutes”[MeSH] OR “Dietary Sodium”[MeSH] OR “Potassium, Dietary”[MeSH] OR salt substitute OR low sodium salt OR reduced sodium salt OR potassium-enriched salt)* AND (“Hypertension”[MeSH] OR hypertension OR high blood pressure) AND (“Incidence”[MeSH] OR incidence OR new-onset hypertension OR development of hypertension).

Equivalent search strategies were adapted for Web of Science and Cochrane Central using the syntax and controlled vocabulary specific to each database. Abstracts, full-text articles, and scientific conference proceedings were screened. Reference lists of included studies and relevant systematic reviews were manually reviewed to identify additional eligible studies.

Initial screening was based on titles and abstracts, with no restrictions on publication date. All retrieved records were imported into the Rayyan^®^platform to facilitate the screening process.^16^During this stage, studies unrelated to the research question were excluded, and duplicate entries were removed. Three review authors (C.R.D, L.E.R.S, and F.B.B) conducted the screening independently, and disagreements were resolved through discussion with a third review author (F.A.K). Automated alerts were also configured in the databases to notify the review team of newly published studies during the analysis period.

### Data extraction

To summarize the main findings, three review authors (F.A.K, L.E.R.S, and F.B.B) independently extracted data from the four included articles. Data included first author’s surname, year of publication, study design, follow-up period, patient characteristics (age, sex, and comorbidities), assessment methods, and conclusions regarding changes in SBP and DBP. Disagreements were resolved by consensus, and a third review author (C.R.D) made the final decision when necessary.

### Endpoints and definitions

The primary outcome of interest was the change in SBP and DBP.

### Assessment of risk of bias

The Revised Cochrane Risk-of-Bias Tool for Randomized Trials^17^was used to evaluate the methodological quality of the included RCTs. Two authors (C.R.D and L.E.R.S) independently assessed all studies, and disagreements were resolved by consensus. Each trial was classified as having a high, low, or unclear risk of bias across five domains: randomization process, deviations from intended interventions, missing outcome data, measurement of outcomes, and selection of reported results.

Potential publication bias and minor study effects were assessed using funnel plot analysis to determine whether the distribution of trials was symmetrical.

### Statistical Analysis

Treatment effects for continuous outcomes were pooled and expressed as mean differences (MD) with 95% CIs. Heterogeneity was assessed using the width of effect sizes and the I^2^and τ^2^statistics. A fixed-effect model was used for endpoints with I^2^< 25%, whereas the DerSimonian and Laird random-effects model was applied for pooled outcomes with higher heterogeneity, considering both I^2^and τ^2^(between-study variance). A p-value < 0.05 was considered statistically significant.

Statistical analyses were performed using RStudio version 4.2.3, with the “metafor” package (R Foundation for Statistical Computing). A sensitivity analysis was conducted to evaluate the impact of individual studies by sequentially removing each RCT and reanalyzing the remaining data (leave-one-out analysis). Study dominance was defined when the removal of a study changed the statistical significance of the pooled effect size p-values, either from significant to nonsignificant or vice versa.

## Results

### Selection of studies and baseline characteristics

As detailed in Figure 1 , the initial search yielded 221 records. After removing duplicates and screening studies based on title and abstract, five studies remained for full-text review according to the prespecified criteria. Of these, four studies were included, comprising 1,430 patients, of whom 725 (50.6%) received the salt substitute. The search strategy is summarized in Figure 1 . Central Illustration presents the main findings of this study.

**Figure 1:** – Preferred Reporting Items for Systematic Reviews and Meta-Analyses flow diagram of study identification, screening, eligibility, and inclusion.

Across the four included studies, 1,430 patients were analyzed, with 725 (50.6%) receiving the salt substitute and 705 (49.3%) receiving regular salt. Follow-up duration ranged from 2 weeks to 2 years. The study population included 777 (54.3%) men and 653 (45.6%) women; 292 (20.4%) had a history of smoking, and 303 (21.1%) reported alcohol consumption. Mean age ranged from 61.5 to 71.8 years, body mass index (BMI) ranged from 23.1 to 31 kg/m^2^, SBP ranged from 121.9 to 177.6 mmHg, and DBP ranged from 74.4 to 105.8 mmHg. Baseline characteristics of each included study are presented in Table 1 .

**Table 1:** – Baseline demographic, clinical, and treatment characteristics of the included studies, presented by study

Characteristic	Barros et al.^**18**^	Zhao et al.^**19**^	Yu et al.^**20**^	Zhang et al.^**21**^
**Number of patients**	IG: 19; CG: 16	IG: 141; CG: 141	IG: 252; CG: 250	IG: 313; GC: 298
**Age, years (SD)**	—	IG: 62.8 (11.1); CG: 63.5 (11.3)	IG: 61.5 (11.1); CG: 61.7 (12.9)	IG: 71 (9.7); GC: 71.8 (10.3)
**Follow-up**	4 weeks	3 months	3 months	2 years
**Male, n (%)**	—	IG: 56 (39.7%); CG: 60 (42.6%)	IG: 105 (41.7%); CG: 102 (40.8%)	IG: 231 (73.8%); GC: 223 (74.8%)
**BMI, kg/m^**2**^(SD)**	IG: 29.38 (5.55); CG: 31 (5.97)	IG: 23.7 (3.1); CG: 23.6 (3.4)	IG: 23.1 (4.7); CG: 23.6 (4.2)	—
**Smoking history, n (%)**	—	IG: 6 (8.2%); CG: 7 (9.7%)	IG: 37 (14%); CG: 34 (13.6%)	IG: 110 (35.1%); GC: 98 (32.9%)
**Alcohol consumption history, n (%)**	—	IG: 8 (11%); CG: 9 (12.5%)	IG: 115 (45.6%); CG: 110 (44%)	IG: 33 (10.5%); GC: 28 (9.4%)
**SBP, mmHg (SD)**	IG: 142.95 (14.86); CG: 143.44 (13.99)	IG: 176.1 (22.4); CG: 177.6 (23.3)	IG: 132.8 (20.3); CG: 132.1 (22.5)	IG: 122 (12); GC: 121.9 (11.1)
**DBP, mmHg (SD)**	IG: 89.79 (9.10); CG: 91.19 (9.10)	IG: 103.2 (12); CG: 105.8 (13.1)	IG: 83.7 (12); CG: 82.9 (13.1)	IG: 74.4 (8.5); GC: 74.5 (7.7)
**Use of antihypertensive drugs, n (%)**	—	IG: 61 (47%); CG: 71 (50.7%)	Diuretic = IG: 0; CG: 1 (0.4%) CCB = IG: 6 (2.4%); CG: 9 (3.6%) ACEi or ARB = IG: 70 (27.8%); CG: 80 (32%) β-blocker = IG: 63 (25%); CG: 50 (20%) α-blocker = IG: 107 (42.5%); CG: 97 (39%)	—
**Salt substitute composition**	IG: NaCl 27.3% + KCl 72.6%; CG: NaCl 100%	IG: NaCl 70% + KCl 30% + MgSO_4_10%; CG: NaCl 100%	IG: NaCl 70% + KCl 30%; CG: NaCl 100%	IG: NaCl 62.5% + KCl 25% + 12.5% of flavorings; CG: NaCl 100%
**History of CVD, n (%)**	—	—	IG: 3 (1.2%); CG: 4 (1.6%)	IG: 56 (17.9%); CG: 67 (22.5%)
**History of diabetes, n (%)**	—	—	IG: 59 (23.4%); CG: 51 (20.5%)	—

All included studies adopted a statistical significance level of 5% (α = 0.05). ACEi: angiotensin-converting enzyme inhibitor; ARB: angiotensin II receptor blocker; BP: blood pressure; CCB: calcium channel blocker; CG: control group; CVD: cardiovascular disease; IG: intervention group; SD: standard deviation.

### Changes in systolic blood pressure

SBP was significantly reduced in the salt substitute group compared with the regular salt group (MD, −5.75 mmHg; 95% CI, −6.98 to −2.39 mmHg; I^2^= 37%; τ^2^= 1.31; p < 0.01) ( Figure 2A ).

**Figure 2:** – Forest plots of MD in changes in BP comparing salt substitute versus regular salt. A) Change in SBP; B) change in DBP. BP: blood pressure; DBP: diastolic BP; MD: mean difference; SBP: systolic BP.

Salt substitution resulted in an approximate 5 mmHg reduction in SBP across the included studies. Reductions of this magnitude have been associated with a 10%-15% decrease in the risk of major cardiovascular events, including stroke and myocardial infarction, which suggests a potential population-level impact on cardiovascular risk.

### Changes in diastolic blood pressure

DBP was significantly reduced in the salt substitute group compared with the regular salt group (MD, −1.62 mmHg; 95% CI, −2.34 to −0.91 mmHg; I^2^= 0%; τ^2^= 0; p < 0.001) ( Figure 2B ).

Although the reduction in DBP was smaller, even modest decreases in DBP have been associated with a lower risk of cardiovascular events, particularly in older adults. This suggests that salt substitutes may contribute to overall cardiovascular risk reduction, complementing the benefits observed for SBP.

Subgroup-specific effects could not be evaluated, as none of the included studies reported outcomes separately for high-risk populations such as individuals with chronic kidney disease or established CVD. Although the observed reductions in SBP (−5.75 mmHg) and DBP (−1.62 mmHg) are clinically relevant at the individual level, the downstream implications for population-wide cardiovascular prevention remain uncertain. Large-scale modeling studies have shown that even small average decreases in BP can lead to meaningful reductions in cardiovascular morbidity and mortality; however, this meta-analysis lacked sufficient data to directly estimate those benefits. Further RCTs with stratified analyses and longer follow-up are required to clarify the true public health impact of salt substitute interventions.

### Assessment of risk of bias

Online Resource Figure 1 and Online Resource Figure 2 summarize the risk of bias for each included study. All four studies were classified as having a low risk of bias, resulting in an overall low risk of bias across the analysis.

The funnel plots for the outcomes showed an asymmetrical distribution of the included studies ( Figure 5 ; Online Supplementary Figure 3 ).

**Figure 5:** – Funnel plot assessing publication bias in the analysis.

### Sensitivity analysis

No study was excluded due to methodological heterogeneity; leave-one-out and Baujat plot sensitivity analyses were performed for all continuous outcomes.

In the leave-one-out analysis for SBP, omitting Barros et al.^18^and Zhao et al.^19^increased heterogeneity to 56% and 53%, respectively. In contrast, omitting Yu et al.^20^and Zhang et al.^21^reduced heterogeneity to 0% in both analyses ( Figure 3A ). For DBP, there was no meaningful change in heterogeneity when individual studies were removed; however, when Zhang et al.^21^was excluded, heterogeneity increased to 13% ( Figure 3B ).

**Figure 3:** – Forest plots of leave-one-out sensitivity analysis for MD in BP comparing salt substitute versus regular salt. A) Change in SBP; B) change in DBP. BP: blood pressure; DBP: diastolic BP; MD: mean difference; SBP: systolic BP.

Baujat plot analysis indicated that Yu et al.^20^had the greatest influence on the overall results for both SBP and DBP analyses. Additionally, Zhang et al.^21^contributed most to overall heterogeneity in both analyses ( Figure 4 ; Online Supplementary Figure 4 ).

**Figure 4:** – Baujat plot for systolic blood pressure.

### Meta-regression analysis

A meta-regression analysis was performed to investigate whether mean age and mean BMI could explain between-study heterogeneity in BP outcomes. For SBP, age showed a borderline association with treatment effect, suggesting a trend toward greater BP reduction in studies including older participants (coefficient: −0.26; p = 0.0557; I^2^= 0%). In contrast, BMI was not significantly associated with the systolic response to the intervention (coefficient: −0.84; p = 0.4535; I^2^= 0%).

For DBP, neither age (coefficient: −0.04; p = 0.7387; I^2^= 29.9%) nor BMI (coefficient: −0.67; p = 0.3388; I^2^= 19.3%) were significant moderators of treatment effect. These findings suggest that the BP-lowering effects of salt substitutes appear consistent regardless of participants’ age or BMI within the ranges reported in the included trials ( Online Supplementary Figure 5 ; Online Supplementary Figure 6 ; Online Supplementary Figure 7 ; Online Supplementary Figure 8 ).

## Discussion

In this systematic review with meta-analysis of four RCTs including 1,430 participants, salt substitutes, typically formulated by partially replacing sodium chloride with potassium chloride and/or calcium, were associated with significant reductions in BP compared with regular salt. Hypertension is the leading global risk factor and contributes to the loss of 174 million disability-adjusted life years annually,^22^and its management remains suboptimal in low- and middle-income countries. Because discretionary salt added during cooking accounts for most sodium intake in these settings, salt substitutes may represent a feasible and impactful intervention.^23 , 24^

The pooled reduction in SBP was −5.8 mmHg (95% CI, −7 to −4.6 mmHg), while DBP decreased by −1.6 mmHg (95% CI, −2.3 to −0.9 mmHg). These findings demonstrate a consistent BP-lowering effect, comparable in magnitude to established antihypertensive interventions, and support salt substitution as a promising strategy for population-level BP control.

Our findings integrate and extend previous research evaluating the effects of salt substitutes on BP. Early investigations conducted before 2010 provided limited evidence of clinically meaningful BP reductions.^21 , 25 - 29^One notable exception was the Chinese cluster RCT by Mu et al.,^24^which demonstrated a 4.2/1.9 mmHg decrease in SBP and DBP over 12 weeks. The SBP reduction observed in our pooled analysis (−5.8 mmHg) is broadly consistent with this early signal and underscores that the hypotensive effect of salt substitutes persists across age groups and cultural contexts.

More recent clinical trials in adults have reported reductions in SBP of approximately 3-6 mmHg, closely matching the magnitude of our overall estimate. Barros et al.^18^observed a 5.6 mmHg greater reduction in SBP with a 50% NaCl/50% KCl preparation over 3 months, while Zhao et al.^19^reported a 4.8/1.7 mmHg decrease after 6 months. Similarly, Yu et al.^20^and Zhang et al.^21^documented SBP reductions of 3-5 mmHg. The consistency between our findings and these trials reinforces the robustness of the effect size and highlights its reproducibility across geographical regions, baseline risk profiles, and formulations of potassium-enriched salt.

Furthermore, the magnitude of SBP reduction observed in this meta-analysis aligns with large-scale epidemiological modeling from the INTERnational study of SALT and blood pressure and GBD studies,^30 - 33^which associate population-level reductions of approximately 5 mmHg in SBP with meaningful decreases in cardiovascular risk. While these estimates provide context for potential public health benefits, they remain theoretical extrapolations rather than direct outcomes measured in the included trials.

Our findings reinforce the potential of salt substitutes as an effective, family-based strategy for the primary prevention of hypertension.^34 , 35^Replacing sodium chloride with potassium chloride lowers extracellular volume while enhancing natriuresis and vasodilation.^36 - 39^From a public health perspective, salt substitutes are particularly suited for low- and middle-income countries, where discretionary salt intake accounts for more than 70% of total intake and industrial reformulation remains limited.^39 - 43^Combining salt substitution with educational strategies aimed at reducing total salt intake to ≤ 3 g/day may further amplify the benefit.^43 - 45^In this meta-analysis, potassium-enriched salt substitutes reduced SBP by a pooled MD of −5.8 mmHg (95% CI, −7 to −4.6 mmHg) and DBP by −1.6 mmHg (95% CI, −2.3 to −0.9 mmHg) compared with regular salt. These reductions are comparable to those achieved with first-line antihypertensive medications.

Despite these encouraging findings, several methodological limitations of the included studies must be acknowledged. First, heterogeneity across studies, particularly for SBP, was moderate, which reflects differences in patient populations, follow-up durations, and intervention compositions. Second, some trials had short intervention periods, limiting the assessment of sustained BP reduction and long-term safety. Third, publication bias cannot be fully excluded because of the small number of RCTs and the relatively narrow inclusion criteria. Fourth, although all included studies were randomized, blinding procedures and compliance reporting varied, potentially introducing performance bias or detection bias. Finally, serum potassium monitoring was inconsistently reported, leaving uncertainty regarding the long-term safety of potassium-enriched salts, particularly the risk of hyperkalemia in patients with impaired renal function or those receiving renin-angiotensin-aldosterone system-blocking drugs.

## Conclusion

This meta-analysis demonstrates that using potassium-enriched salt substitutes is significantly more effective than regular salt in lowering both SBP and DBP among patients with hypertension. These findings reinforce the role of salt substitutes as a practical and low-cost strategy for the prevention and management of hypertension, with the potential to substantially reduce cardiovascular events at the population level.

However, important questions remain unanswered, particularly regarding the long-term safety of these interventions and their hypotensive effects in normotensive individuals, who have often been excluded from RCTs. Future large-scale clinical studies should include these populations, incorporate systematic potassium monitoring, and explore the mechanisms underlying the BP-lowering effects of salt substitutes. Such investigations will be essential to fully characterize their preventive potential and to inform precision strategies in cardiovascular care.

Overall, this meta-analysis supports the role of salt substitutes as a cost-effective, accessible tool to reduce BP and potentially prevent cardiovascular events. Nevertheless, to ensure safe implementation, future RCTs with longer follow-up and standardized potassium monitoring are needed to confirm durability, optimize dosing ratios, and evaluate long-term outcomes, including hyperkalemia and cardiovascular mortality.

## Supplemental Materials

Supplementary Material
